# Successful Delivery of Twin Pregnancy in Class U3b/C2/V1 Uterus by Bilateral Caesarean Section after Spontaneous Conception

**DOI:** 10.1155/2015/743621

**Published:** 2015-10-18

**Authors:** Yasmine El-Masry, Ahmed M. E. Ossman, Mohammed El-Namoury, Sameh Sarsik

**Affiliations:** ^1^Obstetrics and Gynecology Department, Tanta University Hospital, Tanta 31962, Egypt; ^2^Tanta University Hospital, Tanta 31962, Egypt

## Abstract

A case of a 19-year-old female with class U3b/C2/V1 uterus conceived a twin pregnancy with a fetus in each horn after spontaneous conception. She referred to our department with presentation of premature rupture of membranes, with a history of cesarean delivery of a single full term living fetus a year and a half before this delivery. Examination revealed two completely separate uterine horns with a fetus in each horn, two distinct externally rounded cervices, and a single vagina with a short nonobstructing vaginal septum in the upper part of the vagina. And as the appropriate mode of delivery is still unclear, each case should be managed as the condition requires, and in our case urgent bilateral caesarean sections were performed.

## 1. Introduction

In this paper, we are reporting a rare case of delivery of a twin pregnancy in class U3b/C2/V1 after spontaneous conception. A wide variety of uterine anomalies occur as a result of abnormal fusion and canalization of Müllerian ducts, class U3b uterus (formerly, didelphic uterus), results from complete failure of Müllerian ducts to fuse, giving rise to the duplication of the uterine cavity and cervix [1–3]. The prevalence of uterine anomalies is about 5.5% among unselected population as diagnosed by the investigations that can diagnose and classify the uterine anomalies [4]. The incidence of class U3b uterus represents 11.1% of the uterine anomalies [5].

Although U3b uterus has better chance to get pregnant than other uteri with congenital anomalies [6], the outcome of such pregnancies is not favored [7].

These circumstances and an estimated natural twin conception with fetus in each horn of class U3b/C2 uterus of one per million [8] make such cases extremely rare events.

## 2. Case Report

A 19-year-old woman, gravida 2, para 1, living 1, presented to the Obstetrics and Gynecology Department, Tanta University Hospital, for the first time on Monday, December 16, 2013, with a complaint of gush of watery vaginal discharges, persistent progressive low back pain, and tender old caesarean scar. She was examined at the primary health care unit at her village and because of the lack of facilities, she was referred to our hospital.

Gynecological history revealed that her menarche was by the age of 9; she had regular menstrual cycles of average amount every 28 days that last for 3 to 4 days. Her first delivery was in 2012 by cesarean section due to contracted pelvis and resulted in delivery of a full term healthy fetus, and the patient denied any knowledge about her uterine anomaly. After a period of 6 months of lactational amenorrhea, she started contraceptive minipills for a month during which she conceived naturally due to irregular intake of the contraceptive pills.

During this pregnancy, she did not follow her gestational status regularly at antenatal care center. The patient stated that she had recurrent attacks of urinary tract infection over the last few months and episodes of low back pain that subsided by over-the-counter analgesics.

Obstetrical examination revealed two fundal levels at the 36th week of gestation, occupying right and left hypochondrial areas, respectively. By speculum examination, the patient was diagnosed as definite PROM (premature rupture of membranes), and two distinct externally rounded cervices were detected with dilatation of one cervix about 3 fingers, while the other cervix was closed, and a short nonobstructing vaginal septum was found.

Ultrasound showed male and female fetuses at the 34th and 36th weeks of gestation, respectively, with cephalic presentation occupying both uterine cavities.

Doppler showed normal fetal heart sounds that were heard in the right and left iliac fossae with a rate of 130 and 145, respectively.

**Figure 1:**
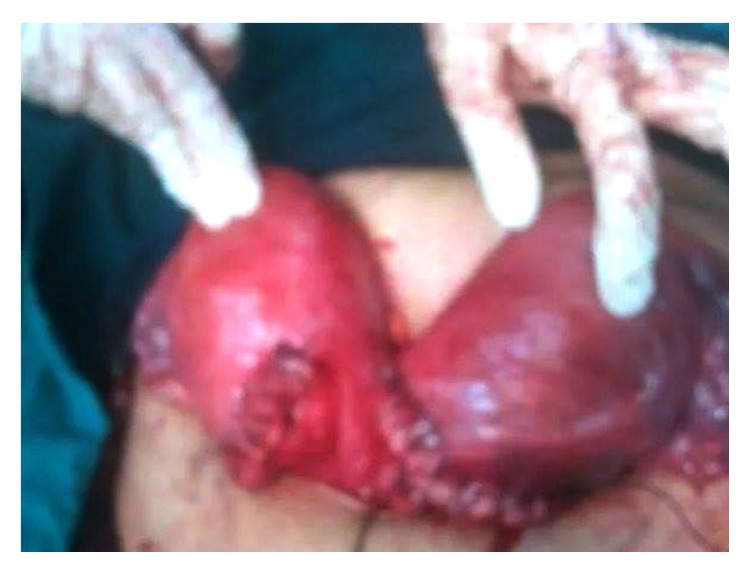
Intraoperative image of the gravid uteri, after delivery of both fetuses by lower uterine caesarean incisions; we started with the right horn and then the left one; the right horn contained a single female fetus; the left horn contained a single male fetus. The uterine incisions were repaired in two layers without any trauma to the bladder or the colon.

Patient underwent urgent caesarean delivery. Intraoperatively, we detected two separate uterine horns with colonic loops and omentum in the gap in between them (Figure 1). We started with the right horn, as it was more accessible and the head of the fetus in the other horn was deeply engaged in the pelvis. Through a low segment incision, a single living female fetus was delivered safely. Immediately, we started with the left horn and a single living male fetus was safely delivered. The uterine incisions were repaired in two layers.

The female fetus birth weight was 2500 gm and her Apgar score was 9/10, while the male fetus birth weight was 2000 gm and his Apgar score was 8/10; both fetuses were transferred to the neonatal intensive care unit for respiratory assistance.

After the surgery, the mother's hemoglobin level was 7 gm/dL, so she was admitted to the inpatient unit and received three packs of blood, one pack of plasma, five ampules of Ferosac (intravenous iron saccharate complex) divided into two doses, antibiotics, and intravenous infusions.

The ultrasonography was repeated and revealed two uterine horns and absence of intra-abdominal fluids, and as the patient's hemoglobin level and health status were improved she was discharged on the seventh day. The rest of the postpartum period was uneventful.

## 3. Discussion

Around the 12th week of female fetus development, the caudal portion of Müllerian ducts fuses to form the uterovaginal canal; this canal is formed of two channels separated by a septum. By the 20th week, reabsorption of the septum starts in the cephalic direction giving rise to the uterus and the upper portion of the vagina. Arrest of development at any point gives rise to various subtypes of class U2 (formerly, septate uterus) and class U3 such as U3a (formerly, bicornuate uterus), while complete failure of Müllerian ducts fusion results in U3b (formerly, uterine didelphys) [1, 3].

Delivery of twin gestation, one in each horn of class U3b uterus, is a rare condition as it is usually associated with complications which make reaching full term challenging such as miscarriage, malpresentation, placental abruption, preterm delivery, and fetal death [7], so a high index of suspicion is needed for early diagnosis of such cases, because about 68% of the patients with uterine anomalies are recognized while in actual labor [9] as in this case, and such cases require close observation during the prenatal period to avoid complications.

A study by [10] recommended bilateral cervical cerclage to be performed between the 12th and 14th weeks of gestation as it helps in the prevention of preterm labor in such cases, but we could not perform this procedure for our case, as it was presented to our department on the day of delivery.

Although a recent study suggested that prediction of the mode of delivery can be done according to the number of uterine cavities and external uterine orifices, for a class U3b/C2 pregnant uterus, caesarean delivery was suggested [11], but reported cases of twin pregnancy with class U3b uterus are uncommon. Therefore, the final decision needs more studies to be conducted, and, until then, each case should be managed as it requires (Table 1).

## Figures and Tables

**Table 1:** The mode of delivery and the outcome of cases of twins with U3b/C2 uterus.

References	Mode of conception	Occupied side of the uterus (right horn/left horn)	Gestational age at delivery (first/second)	Mode of delivery (first/second)	Outcome
Kennedy, 1959 [12]		1/1	35 w + 3/38 w	V/V	Two living neonates
Jones and Flanagan, 1973 [13]	Spontaneous	1/1	34 w	C/C	Two living neonates: a male and a female
Clarke 1977 [14]		1/1	38 w	V/C	Two living male neonates
Kanakas et al., 1989 [15]		2/0	34 w	C/C	Two living male neonates
Kekkonen et al., 1991 [16]		1/1	38 w	C/C	Two living neonates
Ginsberg et al., 1997b [17]	Three eggs transferred to each fallopian tube	2/1	38 w + 1 d	V	Single living male neonate, from the right horn, after gestation reduction
Brown et al., 1999 [18]		1/1	26 w	C/C	Two living neonates: a male and a female
Tyagi et al., 2001 [19]			33 w	V/V with 5-day spacing	Two living female neonates
Mor et al., 2002 [20]		1/1	32 w	C/C	Single living neonate
Nohara et al., 2003 [21]	Induction of ovulation Transcervical insemination	1/1	25 w/35 w	C/V	Two living male neonates
Garg et al., 2010 [22]		1/1	36 w + 4 d	C/C	Two living neonates
Jan et al., 2013 [23]		1/1	35 w + 2 d/38 w + 2 d	V/V	Two living neonates: a male and a female
Jackson et al., 2014 [24]		0/3	29 w	C/C	Three living male neonates
Maki et al., 2014 [25]		1/1	37 w	V/C	Two living neonates
Our case	Spontaneous	1/1	36 w	C/C	Two living neonates: a male and a female

V: vaginal delivery, C: cesarean delivery, numbers: number of fetuses per side, w: week, and d: day.

## References

[1] Reichman D. E., Laufer M. R. (2010). Congenital uterine anomalies affecting reproduction. *Best Practice and Research: Clinical Obstetrics and Gynaecology*.

[2] Taylor E., Gomel V. (2008). The uterus and fertility. *Fertility and Sterility*.

[3] Gordts S. (2013). The ESHRE-ESGE consensus on the classification of female genital tract congenital anomalies. *Gynecological Surgery*.

[4] Chan Y. Y., Jayaprakasan K., Tan A., Thornton J. G., Coomarasamy A., Raine-Fenning N. J. (2011). Reproductive outcomes in women with congenital uterine anomalies: a systematic review. *Ultrasound in Obstetrics and Gynecology*.

[5] Acién P. (1997). Incidence of Mullerian defects in fertile and infertile women. *Human Reproduction*.

[6] Heinonen P. K. (2000). Clinical implications of the didelphic uterus: long-term follow-up of 49 cases. *European Journal of Obstetrics Gynecology and Reproductive Biology*.

[7] Grimbizis G. F., Camus M., Tarlatzis B. C., Bontis J. N., Devroey P. (2001). Clinical implications of uterine malformations and hysteroscopic treatment results. *Human Reproduction Update*.

[8] Ginsberg N. A., Strom C., Verlinsky Y. (1997). Management of a triplet gestation complicated by Uterus didelphys. *Fetal Diagnosis and Therapy*.

[9] Blair R. G. (1960). Pregnancy associated with congenital malformations of the reproductive tract. *BJOG: An International Journal of Obstetrics & Gynaecology*.

[10] Yassaee F., Mostafaee L. (2011). The role of cervical cerclage in pregnancy outcome in women with uterine anomaly. *Journal of Reproduction and Infertility*.

[11] Takami M., Aoki S., Kurasawa K., Okuda M., Takahashi T., Hirahara F. (2014). A classification of congenital uterine anomalies predicting pregnancy outcomes. *Acta Obstetricia et Gynecologica Scandinavica*.

[12] Kennedy N. (1959). A case of twin pregnancy in a double uterus. *British Medical Journal*.

[13] Jones M. M., Flanagan M. C. (1973). Twin pregnancy in a uterus didelphys delivered by bilateral repeat cesarean sections. *Journal of the National Medical Association*.

[14] Clarke G. C. M. (1977). Uterus didelphys with a pregnancy in each horn. Case report. *British Journal of Obstetrics and Gynaecology*.

[15] Kanakas N., Boos R., Schmidt W. (1989). Twin pregnancy in the right horn of a uterus didelphys: a case report. *European Journal of Obstetrics & Gynecology and Reproductive Biology*.

[16] Kekkonen R., Nuutila M., Laatikainen T. (1991). Twin pregnancy with a fetus in each half of a uterus didelphys. *Acta Obstetricia et Gynecologica Scandinavica*.

[17] Ginsberg N. A., Strom C., Verlinsky Y. (1997). Management of a triplet gestation complicated by uterus didelphys. *Fetal Diagnosis and Therapy*.

[18] Brown O., Mahendran D., Lieberman B. (1999). A twin pregnancy in a uterus didelphys. *Journal of Obstetrics and Gynaecology*.

[19] Tyagi A., Minocha B., Prateek S. (2001). Delayed delivery of second twin in uterus didelphys. *International Journal of Gynecology and Obstetrics*.

[20] Mor E., Saadat P., Sokol R. Z., Paulson R. J. (2002). Spontaneous twin gestation after vaginal dilation in a woman with uterus didelphys and bladder exstrophy. *Obstetrics and Gynecology*.

[21] Nohara M., Nakayama M., Masamoto H., Nakazato K., Sakumoto K., Kanazawa K. (2003). Twin pregnancy in each half of a uterus didelphys with a delivery interval of 66 days. *BJOG*.

[22] Garg R., Kwatra A., Bangal V. B. (2010). Rare case of uterus didelphis with full term pregnancy in each horn. *Pravara Medical Review*.

[23] Jan H., Bizrah M., Hamid R. (2013). A case of spontaneous conceived twins in uterus didelphys, with induction and delayed delivery between twins. *Journal of Obstetrics and Gynaecology*.

[24] Jackson J. R., Williams B., Thorp J. (2014). Spontaneous triplets carried in a uterus didelphys. *Case Reports in Women's Health*.

[25] Maki Y., Furukawa S., Sameshima H., Ikenoue T. (2014). Independent uterine contractions in simultaneous twin pregnancy in each horn of the uterus didelphys. *Journal of Obstetrics and Gynaecology Research*.

